# Evaluating laser surface melting of NiCrAlY-APS coating and its effect on high-temperature oxidation behavior of NiCrAlY/YSZ thermal barrier coating before and after surface melting

**DOI:** 10.1016/j.heliyon.2023.e23094

**Published:** 2023-12-01

**Authors:** Mohammad Gavahian Jahromi, Reza Shoja Razavi, Zia Valefi, hamed Naderi, Saeid Taghi-Ramezani

**Affiliations:** Faculty of Materials & Manufacturing Technologies, Malek Ashtar University of Technology, Iran

**Keywords:** Thermal barrier coating, NiCrAlY coating, Laser surface melting, Thickness, Columnar dendrites, Oxidation

## Abstract

This research study was conducted to investigate the laser melting parameters of NiCrAlY-APS coating. High-temperature oxidation was investigated using yttria partially stabilized zirconia (YSZ) ceramic coating. Also, the oxidation behavior of the TBC coating was investigated and studied before to and after laser surface melting of the NiCrAlY coating. Microstructural characterization was done using a scanning electron microscope (SEM), elemental analysis by energy dispersive spectroscopy (EDS), and phase analysis by X-ray diffraction (XRD). Surface melting was then performed in the power range of 150–300 W and scanning speed of 2–6 mm s^−1^. Surface melting was also conducted on the coating using two strategies: single-pass and multi-pass. The obtained results showed that the average melting depth and thickness reduction were directly related to the laser power, while they had an inverse relation with the laser scanning speed. Furthermore, multi-pass surface melting parameters reduced porosity to less than 0.1 %. Roughness measurements also showed a decrease in the coating's surface hardness after surface melting, as compared to the APS coating. The structure consisted of oriented columnar dendrites after melting the laser. The adhesion strength of the TBC coating and laser surface melting coating was at 41 MPa and 53 MPa, respectively. After 200 h of oxidation in the G1504 sample, the TGO layer's growth was decreased; due to the growth of a single oxide layer, it had better oxidation resistance in comparison to the other sample.

## 1 Introduction

Protecting the surface against high-temperature oxidation is a determining factor in the quality as well as life of most parts [[1], [2], [3]]. Nickel-based superalloys are subject to oxidation when operating at high temperatures as the hot-path components of gas turbines [2]. Thermal barrier coating (TBC) can be regarded as one of the most effective and at the same time, cost-effective strategies to increase the working temperature of gas turbines as well as diesel engines, and to enhance resistance to oxidation and possible thermal shocks [[4], [5], [6]]. TBCs are usually a three-layer structure including a bond coat, a top coat and a thermally grown oxide (TGO) layer [6,7]. Thermal spraying methods are considered the most commonly used coating processes [7]. Atmospheric plasma spraying (APS) methods are widely used in the industry [[7], [8], [9], [10]]. TBCs obtained by the APS method lead to the formation of unwanted phases, oxide bands, surface depressions, elevations, porosity and cracking, as well as unmelted particles and layers with different orientations [[11], [12], [13]]. After some time, these coatings are destroyed and cannot function any further. Improving the properties of the interlayer is effective in enhancing the coating's performance. Currently, various studies have been conducted on post-spray operations done on coatings. This process is suitable for modifying the coating surface by applying a high-power heat source produced by a laser beam.

In this regard, Ming et al. [14] investigated the microstructure and thermal shock of NiCrAlY laser coating on the copper substrate. They reported that proper metallurgical bonding between the considered coating and the substrate could lead to thermal shock resistance. Therefore, the resistance to the thermal shock of the NiCrAlY coating that was created by laser could be more when compared to the spraying methods. Also, they showed that the microstructure of the created coating layer had a dendritic structure with directional growth.

Zhu et al. [15] applied NiCrAlY/Al coating on the Inconel 718 substrate by plasma spraying. They then used laser remelting surface treatment to investigate the coating's microstructure as well as its oxidation behavior at 1200 °C. According to these authors, performing laser surface treatment could cause the formation of a dense layer of α-Al_2_O_3_/Cr_2_O_3_ with the thickness of 2.2 μm. Also, it creates a columnar structure free of cracks and holes on the coating surface. They compared NiCrAlY and NiCrAlY/Al metal coatings before and after laser surface treatment, showing that the latter had higher oxidation resistance following laser surface treatment. Also, performing laser surface treatment on the metal layer reduced the porosity, increased the adhesion strength between the splats, and improved the uniformity of the coating's chemical composition.

Further, Sidhu et al. [16] used surface melting operation for the purpose of reducing the porosity and surface properties of APS stellite 6 coating and NiCrAlY coating. The cross-section evaluation of the coating after surface melting showed a significant reduction in coating porosity to less than 0.5 %. The coating thickness and porosity percentage were decreased after surface melting. The researchers stated that the evaporation of some metals from the surface during remelting was another factor easing the coating thickness. Besides, surface melting could lead to the removal of microstructural inhomogeneities (e.g., unmelted particles and oxidized impurities) and improve the bonding of the coating to the substrate.

In another study, researchers [17] investigated the effect of scanning speed and laser power on the remelted volumes width, melting depth and coating thickness. They stated that laser surface melting led to uniformity in the chemical composition and removed the porosity in the coating. The thermal capacity could be one other cause of change in melting depth, and it is the energy required to melt the coating. In a thick coating, the absorbed energy is stored in the surface layer. Therefore, the greater the thickness of the coating, the higher the melting depth of it. The penetration depth and width of the surface melting zone are linearly related. The correlation coefficient obtained for the samples was between 0.92 and 0.95, thus indicating the accuracy and correctness of the results. Previous research also shows that the width and depth of the single-pass layer are correlated in a linear manner with the laser parameters (i.e., speed and power). According to these researchers, due to the decrease in energy, the width of the single-pass layer is increased with a reduction of the laser scanning speed. Moreover, the width of the single-pass region at constant speed is raised with increasing energy.

Shoja Razavi [18] also applied the coating of stellite 6 by the HVOF spraying method on the 316 L stainless steel substrate. Further, they investigated the oxidation behavior of the stellite 6 coating by performing the surface melting operation to modify the microstructure and improve the mechanical properties. Using the 200 W laser power with a beam scanning speed of 4 mm s^−1^ provided the optimal conditions for multi-pass surface melting operations on the coating and production of ST-Glazing coating. According to this author, performing the surface melting operation could significantly reduce the porosity from 2.3 % to less than 0.3 %. Observations have also shown that performing the surface melting process on the coating of stellite 6 could create a uniform structure and a tremendous amount of Cr_7_C_3_ and (CO, W)_6_ C, as compared to the thermal spraying coating. Melting the coating of stellite 6 caused an increase in hardness, a change in the size of dendritic grains and a decrease of the residual stress. As a result, microhardness was decreased by 17 % (from 625 to 519 Vickers).

In another study [19], high-current pulsed electron beam radiation was used to evaluate the oxidation resistance of NiCrAlY metal bonding coating. In this research, the coating was applied on the surface of the substrate using low-pressure plasma spraying (LPPS). Next, a pulsed electron beam with different current densities was used to melt and modify the coating surface. Afterward, the surface melted sample was subjected to an oxidation test at 900 °C for 200 h. Electron beam irradiation reduced the roughness of the coating surface and consequently, decreased the thickness of the TGO layer. Also, the XRD results showed that the melted surface coating mainly contained α-Al_2_O_3_ and NiO phase.

In a research, laser remelting of NiCoCrAlYSiHf coating was performed through femtosecond laser polishing with high pulse frequency (FLPHPF). Observations showed that laser remelting reduces roughness and changes the distribution of alloy elements. The regenerated and polished melt layer contributes to a thinner, continuous and uniform scale of α- Al_2_O_3_during the oxidation process, they stated that this process is an effective and practical method to improve the high temperature oxidation resistance of MCrAlY coatings [20].

The modification of AIP-NiCoCrAlYSiHf coating plays an important role in increasing the resistance to high temperature oxidation. This process includes surface optimization of the coating, increasing the nucleation rate of Al_2_O_3_ oxides, improving the adhesion stability of Al_2_O_3_, accelerating the lateral growth of Al_2_O_3_ and reducing the rumpling of Al_2_O_3_. Laser radiation to the coating surface has excellent resistance to high temperature oxidation [21].

In the present study, the use was made of NiCrAlY coating on the Hastelloy X substrate by the APS method. The surface melting process was performed using a continuous fiber laser with the power of 1 KW. In the following, the effect of different parameters including laser scanning power and speed on reducing the thickness of the coating and volumes of the melted was investigated. As the next step, optimal parameter, microstructure, hardness and adhesion strength were investigated. Finally, the ceramic coating was applied to the bond coating by using the APS method. The produced melt was evaluated for oxidation resistance at 1100 °C. Overall, this method was expected to improve the final quality of the NiCrAlY-APS coating and enhance the final performance of TBC system coatings, in addition to being affordable.

The innovation of this research is modifying the surface of the middle layer of NiCrAlY-APS by using a fiber laser beam. Investigations showed that laser surface modification could prevent the loss of aluminum, ultimately leading to the improvement of oxidation resistance in thermal barrier coatings.

## 2 Materials and methods

**Fig. 1:**
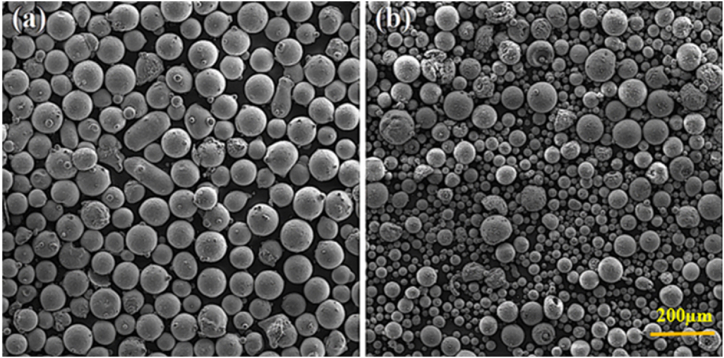
Electron microscopic image a) of NiCrAlY metal powder and b) of the YSZ powder.

In microstructural and phase studies, AISI 420 stainless steel was used to serve as a substrate. The oxidation test is sensitive to the substrate. Thus, Hastelloy X superalloy substrate was used in the oxidation test. The chemical composition of the substrate using spark spectrometry analysis is presented in Table 1. The adhesive strength test was performed according to the ASTM 01–633C standard. To this end, cylindrical samples with the diameter of 25 mm and height of 40 mm were prepared. Peening was performed to eliminate the oxides resulting from the wire-cutting process, create roughness on the substrate's surface, and adhere the considered coating to the substrate. Amdry powder 962 wt% of Y1–Al11–Cr22–Ni was also used to create a NiCrAlY coating as a metal coating connection. Also, Y_2_O_3_ powder (8 wt%) ZrO_2_ with the brand name PAC 2008 was used for a ceramic coating with a spherical morphology in the atmospheric plasma spraying process. Fig. 1a, shows the SEM image of the Amdry metal powder and Fig. 1b Amdry Ceramic powder The specifications of the metal and ceramic powder are given in Table 2. To correctly connect the considered coating to the substrate, we subjected the samples' surface to peening with Al_2_O_3_ particles having a mesh size of 24 μm, angle of 90°, pressure of 5 bar, and distance of 20 cm. Next, the remaining particles were removed from the surface of the samples by air jet and washed with acetone to prevent contamination. NiCrAlY powder was then layered on the surface of the substrates by using atmospheric plasma spraying (APS), model S3000A. This device was equipped with a 4F plasma gun and a 2-10 TW powder injection system made by Sulzer Metco. In this device, all coating parameters could be controlled and applied by computer. A rotary table and a reciprocating system, respectively, could control the movement of the part and plasma torch. In this process, argon and hydrogen are the primary as well as secondary gases for plasma formation, respectively. The optimal parameters of metal coating layering are presented in Table 3.

**Table 1:** Spark spectroscopy analysis of Hastelloy X superalloy.

Ni	Cr	Fe	Mo	Co	W	Si	C	Mn	Al	S	Ti	B	elemnt
48.80	21.47	17.50	8.98	1.23	0.67	0.49	0.095	0.42	0.059	0.018	0.13	0/005	**Wt%**

**Table 2:** Specifications of the NiCrAlY and YSZ powder.

Grain size (μm)	Shape	Chemical Composition	Type of powder
11–125	Spherical	Ni–22Cr–11Al–1Y	Metal
53–106	Spherical	ZrO_2_(wt%)Y_2_O_3_	Ceramics

**Table 3:** Lamination parameters of the NiCrAlY and YSZ powder.

YSZ	NiCrAlY	Unit	Parameter
550	550	A	Current
30	75	l/min	Argon gas flow rate
10	11	SLPM	Hydrogen gas flow rate
3	2.5	l/min	Powder carrier gas flow rate (argon)
35	15	g/min	Powder feed rate
70	120	Mm	Spray distance

**Fig. 2:**
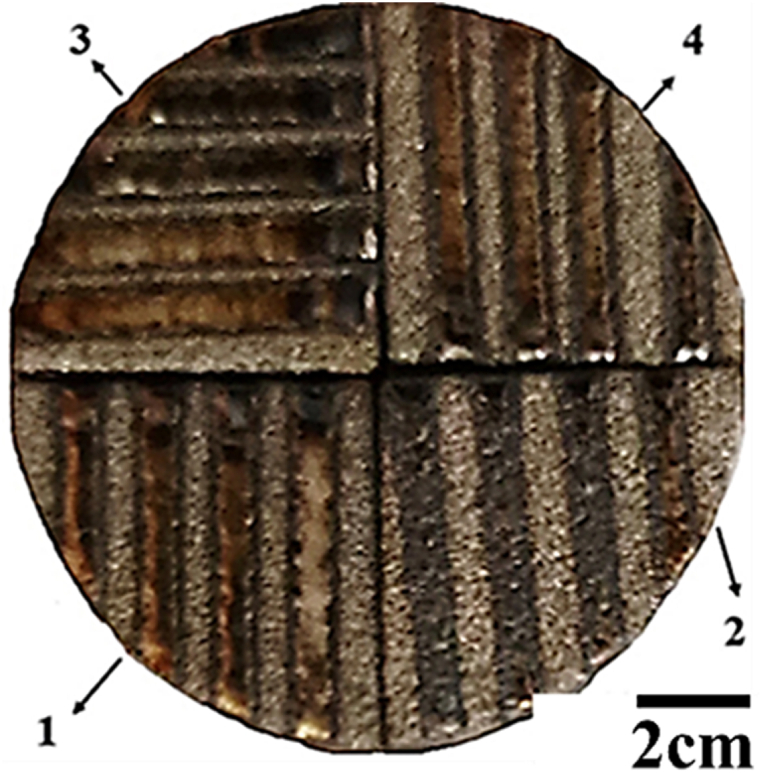
The image of the surface melting of the coating in a single pass.

**Fig. 3:**
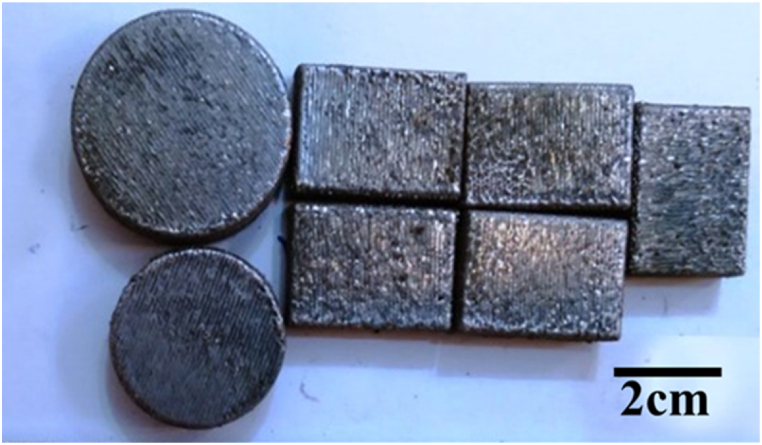
An image of the NiCrAlY coating surface of laser surface melting in full.

**Fig. 4:**
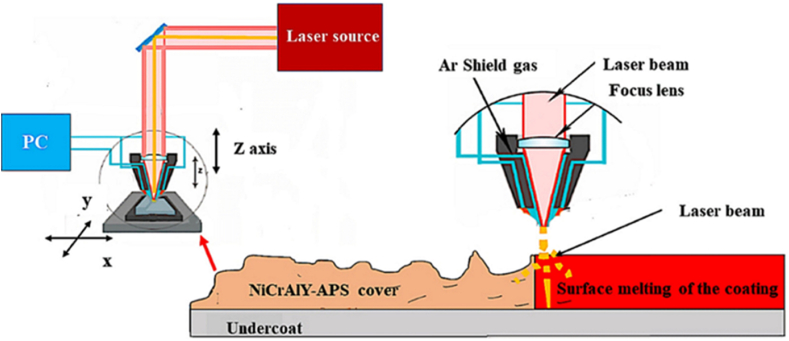
Schematic of laser surface melting.

The surface of the NiCrAlY coating was melted by using a continuous fiber laser with the maximum power of 1 kW. The laser machine had a four-axis Computer Numerical Control (CNC). The parameters which were applied to achieve the best parameters for laser surface melting of the coating are given in Table 4. Laser surface melting parameters were optimized by two strategies including single-pass and multi-pass. The single-pass stage was applied using 15 different parameter groups (Table 4). Also, in the complete melting stage of the coating surface (Table 5), laser surface melting with 50 % overlap was applied to the coating. The samples obtained in this step were named single-pass GL(i) and multi-pass G(i). In regard to multi-pass parameters, (i) represents four numbers. The left side of the first three numbers represents the laser power in watts (W), and the last number denotes the laser scanning speed. For example, G1504 represents 150 W power and 4 mm s^−1^ is the scanning speed. Fig. 2, Fig. 3 illustrate the images of the melted coating in a single pass and the complete melting of the coating, respectively. Also Fig. 4 shows the schematic of the surface melting of the coating.

**Table 4:** Parameters of the single pass surface melting of the NiCrAlY coating.

GL(13–15)	GL(10–12)	GL(7–9)	GL(4–6)	GL(1–3)	Unit	Parameter
300	250	200	180	150	W	Power
4–6					mm/s	Scanning laser

**Table 5:** Parameters of several surface melting passes of the NiCrAlY coating.

G(2506)	G(2006)	G(1806)	G(1504)	Unit	Parameter
250	200	180	150	W	Power
6	6	6	4	mm/s	Scanning laser

In microstructural studies, the samples were subjected to metallographic preparation. Metallographic investigations included polishing of the cross-section. The cross-section images of the considered coatings were prepared by performing metallographic practice according to the ASTM E 1920-03 standard. In this process, the coating may be separated from the sample as a result of the pressure caused by polishing. Therefore, to protect the coating and facilitate better preparation for the next steps, the samples were put in cold-mount epoxy resin; their surface was smoothed by automatic polishing (up to 4000 grit). According to the ASTM-E 2109 standard, to accurately check the porosity and oxide of the coated sample, an optical microscope image was prepared from 10 different parts of the coating to prevent the overlap of the images. Then, the amounts of porosity and oxide were evaluated using ClimaxSiuite. Further, the samples were subjected to structural studies. The microstructure and morphology of the coatings were studied using an Unimet Union8799 optical microscope, backscattered electron images, and a scanning electron microscope manufactured by VEGA TESCAN 410-S. The composition's elemental analysis was performed using an energy dispersive spectrometer of the SAMX device installed on a VEGA TESCAN scanning electron microscope (SEM). The coatings were analyzed before and after surface melting by using an X-ray diffractometer (XRD; AW- XDM 300 made in China) with the wavelength of 1.5406 Kα, voltage of 40 kV, and current of 30 mA. In this research study, the diffraction angle 2θ, scanning time, and step size were considered to be between 20 and 90, 1 s and 0.03°, respectively. Phase identification of the X-ray diffraction patterns was also performed using the X'Pert HighScore software. The surface roughness related to the sprayed as well as lasered coatings was measured by using a Mitutoyo, model P201-SJ, machine made in Japan. Here, Ra, Rq and Rz were reported as roughness numbers. These parameters are the average roughness of three samples with a scanning distance of 5 mm.

The adhesion strength of the coated samples was checked by performing the adhesion strength test, according to the ASTM-C 633-01 standard. The thickness of the TGO layer is considered a measure of oxidation resistance. In this research, 4 series of samples were selected from each parameter. They were then placed in a furnace at 1100 °C after surface melting NiCrAlY coating and applying the YSZ coating for 50, 100, 150, and 200 h. Next, once the samples' cross-sections were prepared, the TGO layer's thickness was measured using an electron microscope, and a measure of the coating behavior in high-temperature oxidation was considered. Eventually, hardness was measured by applying a Vickers hardness tester.

## 3 Results and discussion

In this study, an image and an SEM graph were prepared from the polished cross-section of the coating to examine the thickness as well as structure of the NiCrAlY coating which was applied by the atmospheric plasma spraying process. The results showed a layered structure with melted, unmelted and semi-melted particles, porosity, and alumina particles resulting from atomization. The splats in the coating were separated from each other by alternating oxide layers. Another characteristic of this coating is the oxide veins, which are often parallel to the substrate's surface. The NiCrAlY coating's thickness was measured to be 220 ± 10 μm. During the spraying process, the low kinetic energy of the particles reduced the plastic deformation, eventually creating porosity in the coating structure. There were also irregularities in the coating surface. These irregularities are important in creating a mechanical lock and improving the adhesion of the coating layers [22].

**Fig. 5:**
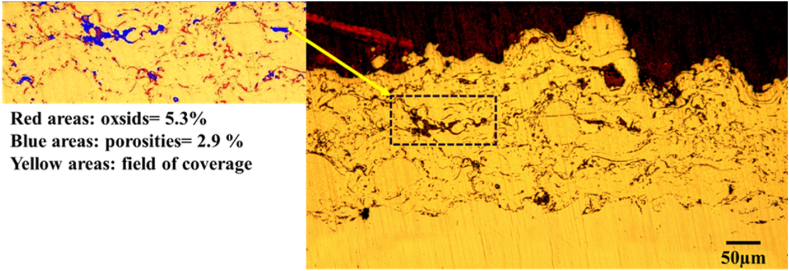
Optical microscopy image of the cross-section of NiCrAlY coating using the clemex software.

According to Fig. 5, porosity could be seen in the coating; it could be justified by examining the characteristics of the plasma spraying process. The porosity is an important and decisive factor in determining the coating's resistance to wear, corrosion and oxidation. Dense coatings with low porosity show better resistance to corrosion and oxidation. The applied coating had 8.6 ± 1.2 % porosity and oxide.

**Fig. 6:**
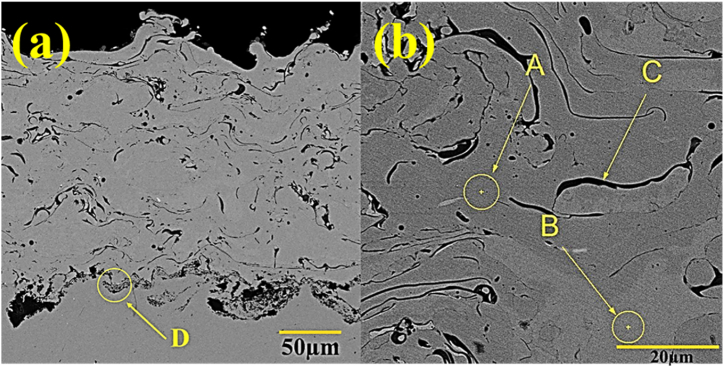
a) Backscattered electron microscopy image of the NiCrAlY coating applied by the APS method at 700× magnification and b) Image related to 1500× magnification.

The three regions A, B, C and D, which are related to the NiCrAlY coating, are shown in Fig. 6. According to energy dispersive spectrum analysis in Table 6, region D shows the analysis of the junction of the bonding coating and the substrate (Fig. 6a). Black islands also confirm the alumina particles remaining from the sand blasting operation. These residual particles are used to increase surface roughness before coating in the substrate preparation process.

**Table 6:** The results of energy dispersive spectroscopy analysis for the points A, B and C in Fig. 6.

Element (wt%)						Area
Fe	O	Y	Al	Cr	Ni	
–	–	0.8 ±0.1	10 ±0.2	22.5 ±0.3	66.7 ±0.3	A
–	34.2 ±0.7	17.9 ±0.6	43 ±0.6	2.5 ±0.2	2.4 ±0.2	B
–	1.6 ±0.2	0.7 ±0.3	6.1 ±0.3	36.4 ±0.2	55.2 ±0.3	C
0.2 ±0.1	47.4 ±0.6	–	51.8 ±0.6	–	0.6 ± 0.2	D

In Fig. 6b, region A of the coating composition, region C of the oxide filament, and region B of unmelted particles and porosity could be seen, respectively. Oxide strands were created on the coating's surface due to powder particles oxidation during the considered process. This has also been reported by other researchers in the spraying path, showing that a layer of oxide could be formed due to the chemical reactions between the surface of the liquid phase and oxygen on the molten particle. The impact of this particle on the substrate surface leads to its expansion. Due to the impact and rapid solidification, the oxide layer is spread under the metal spalts. When applying NiCrAlY coatings, aluminum and yttrium elements have a great tendency to combine with oxygen. Also, molten particles or splats tend to absorb oxygen, forming oxide streaks in the coating. The obtained results are consistent with those of other studies [23,24].

**Fig. 7:**
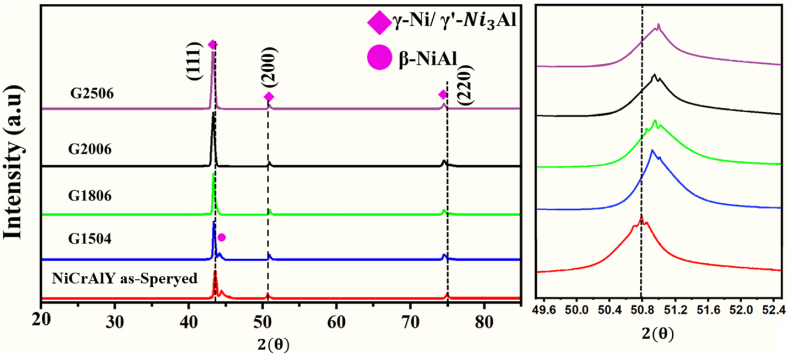
XRD pattern of the coating before and after laser melting.

Fig. 7 shows the XRD pattern before and after laser melting. The main coating was mainly made of γ-Ni/γ′-Ni3Al phases and small amounts of the β-NiAl phase. After surface melting, there was no β-NiAl phase. In laser melting, β phase was converted to γ/γ′ and there was the Al element in the background. After laser melting, peaks (111) and (220) moved to a lower angle (left side), while peak (200) moved to a higher one (right). Residual stress and modification of solute content are two factors changing the peak angle.

**Fig. 8:**
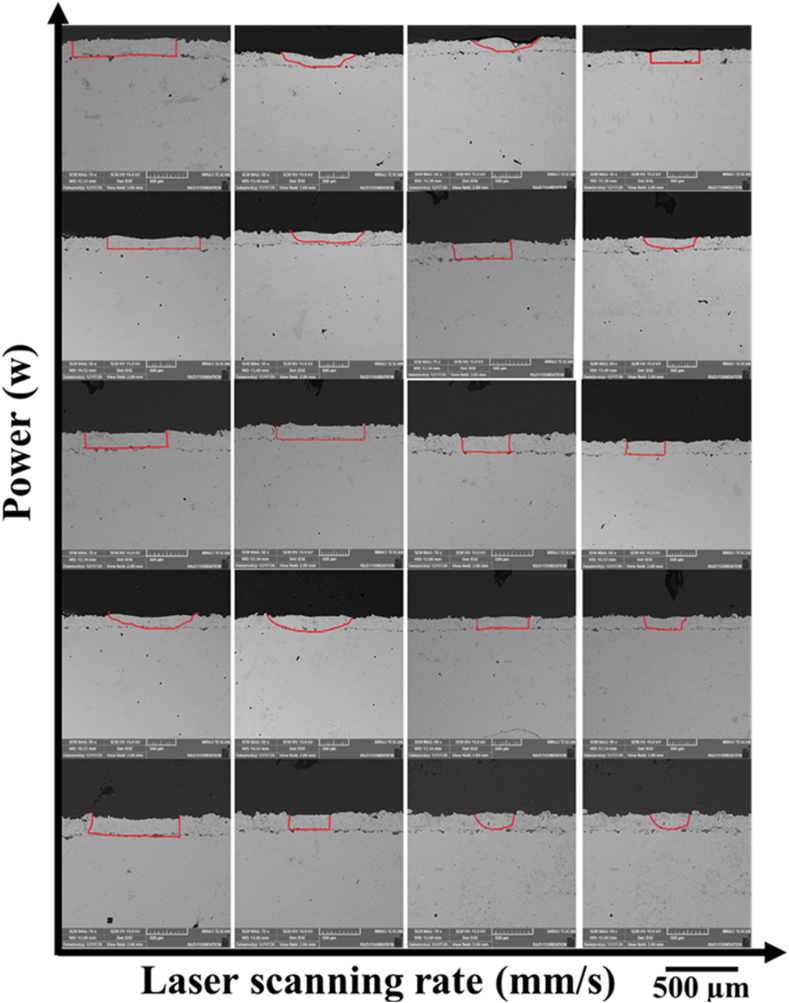
Electron microscopy images of the surface melting volumes in single pass.

**Fig. 9:**
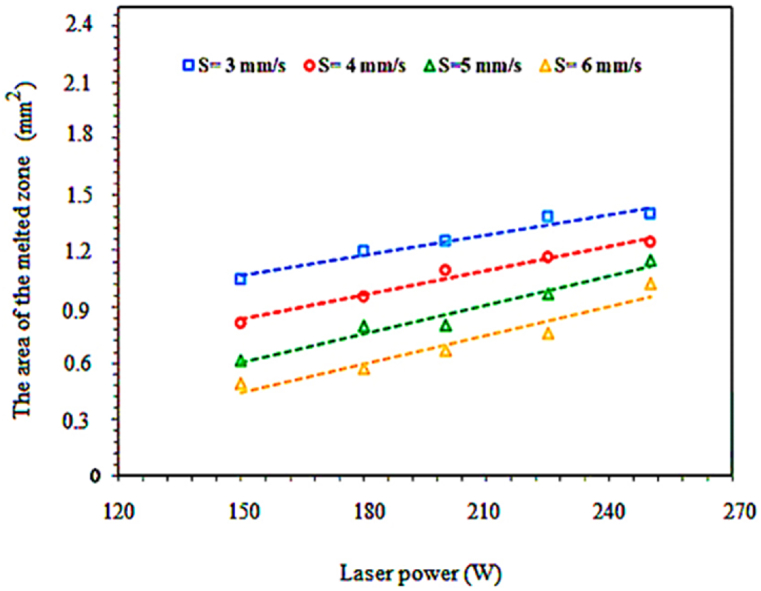
Changes in the volumes or width of the surface melting zone in a single pass according to the laser power.

Single-pass laser surface melting was performed using 15 parameters (Table 4) on the APS-NiCrAlY coating. Scanning speed and laser power are changed in the range of 4–6 mm s^−1^ and 150–300 W, respectively. The best conditions for multi-pass surface melting were selected from the parameters of single-pass surface melting by measuring the melting depth and surface quality (no cracking and change in the coating surface). Fig. 8 shows power changes versus laser scanning speed for the width or volumes of the surface melting (changes are marked with red lines). According to the horizontal graph, from the bottom to the top, the volume of the melted area was raised with increasing power. Also, in the vertical direction, from left to right, the volume of the melted area was decreased with the increase of the laser scanning speed. According to Fig. 9, the volume of the melted area has a direct relationship with the laser power. It also has an inverse relationship with the laser scanning speed. These factors are highly effective in choosing and using laser scanning power and speed. Raising the power from 150 W to 250 W increased the volumes of the surface melting. The largest volume of the melted coating was obtained with the power of 250 W and speed of 3 mm s^−1^. As can be seen, the width of the surface melting zone at a constant power was decreased with increasing the speed from 3 mm s^−1^ to 6 mm s^−1^. Therefore, in the laser surface melting method, the volume of the surface melting could have a linear relationship with the laser scanning power and speed. The obtained results were consistent with the previous investigations as well.

**Fig. 10:**
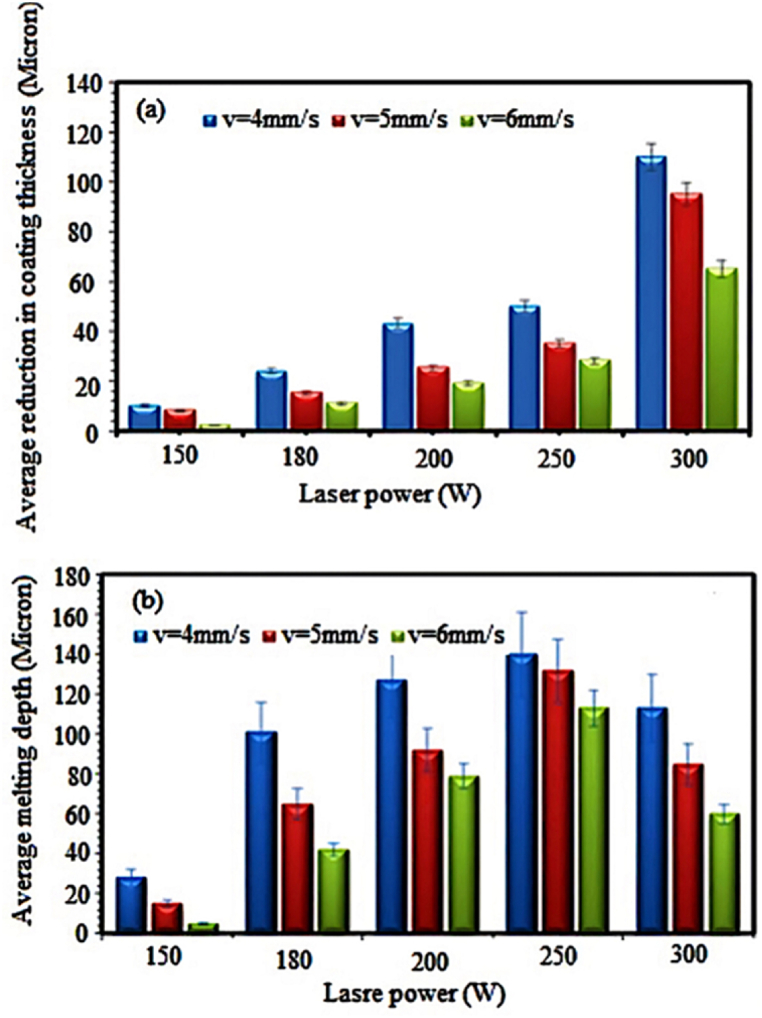
a) Decrease of coating thickness according to the laser power and speed, and b) average depth of the surface melting layer of the metal coating.

Free electrons were excited in the metal coating when the surface was irradiated by the laser beam. This excitation energy was quickly converted into heat. In the following, it was consumed by heat transfer processes, such as conduction in materials, transfer, and radiation from the surface. The most important heat transfer process is thermal conductivity in materials. If the laser beam hits the surface high enough, the laser energy absorption can cause phase transformations, such as surface melting and evaporation. Fig. 10a and b represents the effects of laser interaction with the NiCrAlY metal coating. With increasing the energy density, the melting depth of matter did not increase to infinity (Fig. 10a). The explanation is that when the material's surface temperature reached the boiling point, the melting depth got its maximum value, and the evaporation of the material began (Fig. 10b).

**Fig. 11:**
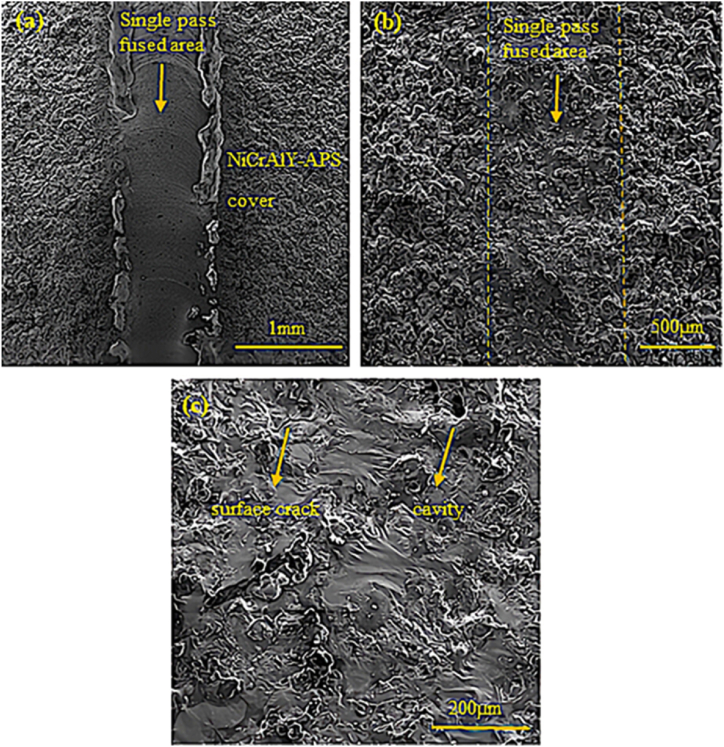
Scanning electron microscopy images of the top surface of the NiCrAlY-APS coating melted by laser in a single pass, a) sample with the code GL1504, b) sample with the code GL2004, and c) sample with the code GL1805.

Fig. 11 shows the SEM images of the surface of the surface-melted NiCrAlY-APS coating in a single pass. Fig. 11a and b also compare the effect of 150 W and 200 W at the scanning speed of mm/s. As can be seen, raising the power from 150 W to 200 W caused the complete melting of the coating surface in the single-pass mode. Fig. 11c also shows the SEM image related to the surface of the surface-melted NiCrAlY-APS coating corresponding to 1805 GL. By increasing the laser power, irregular surface cracks were created on the coating surface. These cracks, which were perpendicular to the coating surface and scattered, were accompanied by surface holes. These holes were due to the release of gas in the porous areas and the displacement of alloy elements during the surface melting operation.

**Fig. 12:**
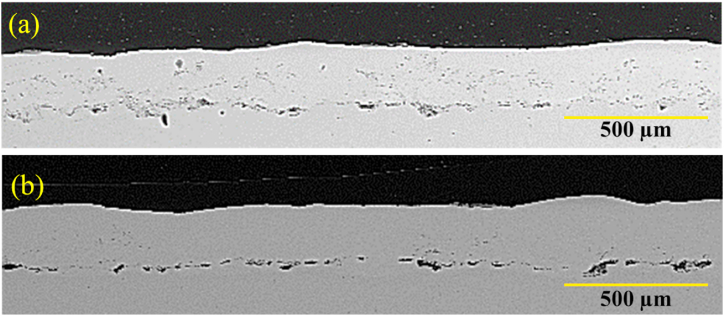
Backscattered electron microscopy images of the laser surface melting NiCrAlY coating related to a) sample with the code G1504 and b) sample with the code G1806.

Fig. 12 presents SEM cross-section images of the melted coating in multi-pass codes G1504 and G1806. As can be seen, the structure of the NiCrAlY coating was altered slightly after laser surface melting. Laser surface melting removed oxide streaks and porosity. After melting in several passes, the surface roughness in the coating disappeared. In Fig. 12a, the depth of melting coating was obtained to be, on average, about 65 μm. In comparison, in the sample G1806, by raising the power from 150 W to 180 W, the melting depth of the coating reached around 140 μm (Fig. 12b). The results, thus, showed that the thickness drop in G1806 was greater than that of G1504. Increasing laser power lowered porosity and released trapped gases in the coating. This phenomenon further decreased the coating thickness in G1806. In this sample, displacement caused by surface tension had occurred. In this case, a large temperature gradient was created on the molten pool's surface. Hence, this caused the non-uniformity of the surface tension in the molten pool. The surface tension occurring in the molten pool center was low, while it was high at the edge of the molten pool. In this case, the two external forces and the reverse flow in the inner part of the melt could reduce the depth of the considered melt pool. Overall, the solidification speed is fast in the laser surface melting process. Therefore, this melting and solidification could lead to elevation loss on the coating surface. The obtained results were, therefore, consistent with those of the previous studies [24,25].

**Fig. 13:**
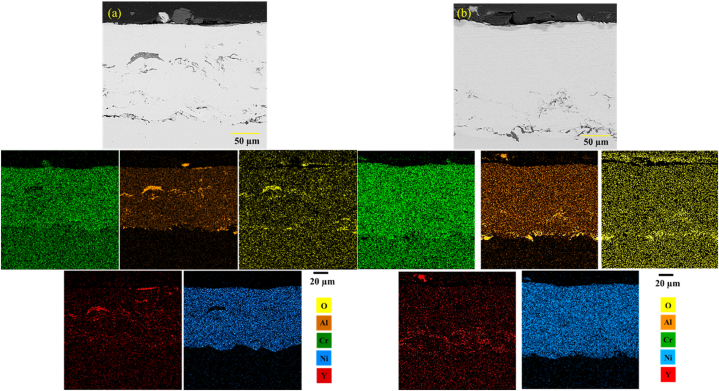
Element distribution map of the cross-section of the surface-melted NiCrAlY coating in a multi-pass manner: a) sample with the code G1504 and b) sample with the code G1806.

The uniformity of the chemical composition in the surface melting zone was studied by performing an element distribution map analysis. Fig. 13a and b shows the results of the element distribution map related to the NiCrAlY surface melting coating. The results showed that the surface melting volumes had a uniform chemical composition of the coating elements. In these two images, the surface melting was increased with the rise of laser power.

Fig. 13a and b illustrate the backscattered electron microscopy images of G2006 and G2506 after laser surface melting. As can be seen, the amount of surface melting was increased with the rise of laser power. In addition, Fig. 13a shows that the coating melting was scattered and around 50 μm in G2006. Finally, according to Fig. 13b, the NiCrAlY coating surface was completely melted by increasing the laser power to 250 W at a constant speed.

**Fig. 14:**
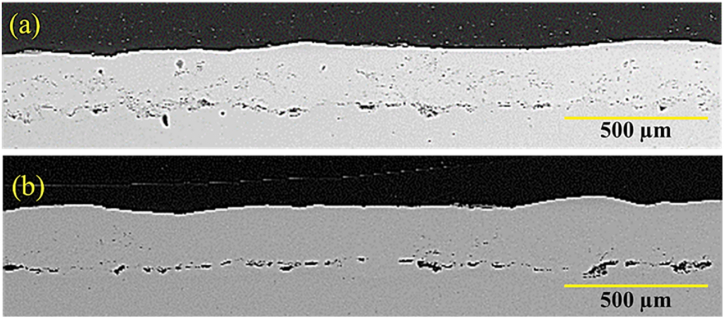
Backscattered electron microscopic images of the laser surface melting NiCrAlY coating related to a) sample with the code 2006G and b) sample with the code G 2506.

**Fig. 15:**
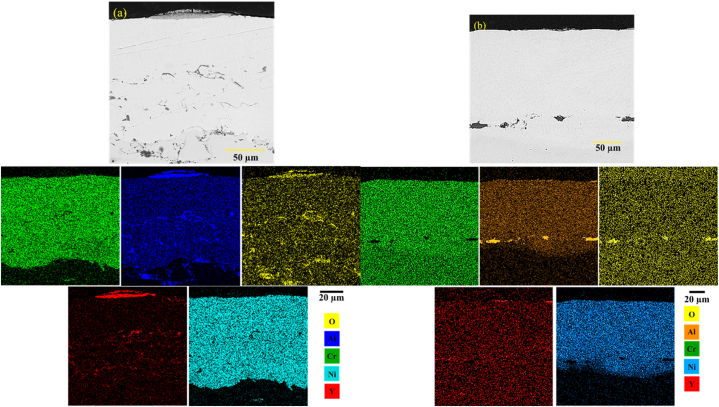
Element distribution map of the cross-section of the surface-melted NiCrAlY coating in a multi-pass manner a) sample with code G2006, b) sample with code G2506.

Fig. 14 shows the distribution map of the elements related to the multi-pass surface melting coating of G2006 and G2506. In backscattered electron microscopy images, light elements have been displayed as gray, and the heavy ones are shown as light (Fig. 14a). According to the backscattered electron images as well as the distribution of the considered elements map, a layer of Al and Y oxides could be seen to be scattered on the coating surface in both samples. As shown in Fig. 14b, after laser melting in G2506, the coating surface was dense and uniform in terms of chemical composition and free of any porosity and oxide streaks. During laser surface melting, the coating surface was protected by the argon gas to prevent oxide formation. Aluminum and yttrium elements were identified in the elements distribution map for the samples G2006 and G2506 in Fig. 15a and b after laser melting. NiCrAlY coating contains different elements with different densities. Aluminum > yttrium > chrome > nickel had the highest density at their melting point, according to their appearance. Creating heat in the NiCrAlY coating to melt the surface by the laser beam caused Al and Y to penetrate from the NiCrAlY coating to the outside surface. The areas formed by the oxide phase had not been emptied due to surface melting, and other elements (e.g., nickel and chromium) were in the section of the formed oxide layer. Accordingly, these elements were not oxidized, and the formed oxide layer was protective, preventing the penetration of oxygen and oxidation of the NiCrAlY coating. This has also been reported by other researchers in similar operations [26,27].

The Y affinity with oxygen outweighs that of Al. However, based on the preferential oxidation, aluminum oxide could always be observed on the surface of the coating due to faster penetration as well as a higher amount of aluminum. Thus, during surface melting, aluminum oxides were formed first. With the continuation of the process of surface melting and heating in the coating, yttrium penetrated through the grain boundaries, forming a thin scattered layer of Y_2_O_3_ on the Al_2_O_3_ layer. In terms of thermodynamics, the formation energy of different elements occurs according to Eq. (1) [12].

(1) $$\left[{\Delta G}_{Y}<{\Delta G}_{Al}<{\Delta G}_{Cr}<{\Delta G}_{Ni}\right]$$

**Fig. 16:**
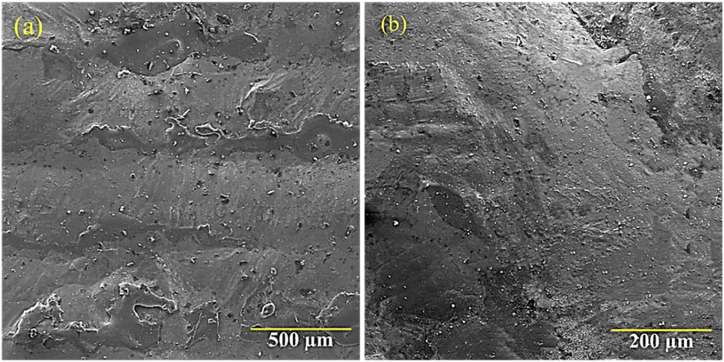
Scanning electron microscopic image of the top surface of NiCrAlY coating after the surface melting of the sample G2506, a) Low-magnified SEM, b) High-magnified SEM.

During laser surface melting, aluminum and yttrium elements moved up under the influence of gravity (at the solid-melt interface), while the heavier metal moved down. Therefore, friction (displacement) or rotation could occur inside the molten pool. When the laser beam hits the surface, heat transfer is done by conduction, convection, or radiation. Convection, the flow of melt (fluid), may be caused by external processes (gravitational fields). In this case, the melt expands unnaturally by the gravity force. In comparison, in the natural state, heat convection is partially transferred by diffusion. The density difference between the two points in the molten pool can be regarded as the main factor in heat transfer due to convection or displacement. Temperature changes lead to differences in density between two points in the melt. As a result, the melt flow is created between two points. The flow of materials in the molten layer is not only caused by the resulting vapor pressure at the point of contact of the beam with the work surface, but also by the surface tension resulting from the thermal gradient in the molten layer [28,29]. Based on the mentioned materials, the melt rotation seems to be another reason for the presence of the oxides of the elements Y and Al. One of the functions of metal bonding coating is to protect the substrate from oxidation. Accordingly, cracks appearing on the top surface of the bond coating indicate the destruction of the coating during oxidation, thermal shock, and adhesion strength tests. By irradiating the laser beam to the surface of the coating, the sample was melted. Also, the melted coating surface was quickly frozen when the laser beam passed through, eliminating the unevenness of the surface and leading to the formation of a surface with low roughness. The two main criteria in the selection of the appropriate parameters in the related laser surface melting process of the metal coating include 1) the decrease of surface roughness and 2) the absence of cracks and holes on the coating's surface. According to Fig. 16a, the coating surface was smooth, dense, and free of cracks and holes. Generally, creating a smooth and dense surface is caused by the rapid melting and solidification of the coating during laser surface melting (Fig. 16b).

Roughness measurement confirmed the smoothness of the coating surface after laser melting. The results of the surface roughness measurement of the NiCrAlY coating before and after laser surface melting in several passes are presented in Table 7. Based on these results, laser surface melting reduced the surface roughness of the coating. The significant decrease in roughness was due to the reduction of unevenness, surface depressions and elevation.

**Table 7:** The results of the roughness measurement of the surface of the laser-melted coating in micrometers (±0.3).

Roughness			Sample code
Rz	Rq	Ra	
26.223	16.521	8.351	NiCrAlY-APS
8.334	4.051	2.415	G(1504)
7.631	3.823	2.115	G(1806)
7.213	2.602	1.810	G(2006)
7.125	1.639	1.502	G(2506)

Clemex, version 3.5.25, software was used to check the amount of oxide and porosity in the laser melting of the APS coating. In fact, based on the ASTM-E2109 standard, 10 optical images with the same magnification (200×) were prepared from the cross-section of each sample, so that the images did not overlap, and their average oxide and porosity values were calculated, as shown in Table 8. Among surface melting samples, G2506 had the lowest porosity due to the complete melting of the coating. The results of measuring porosity in 10 different areas of the coating showed that porosity and oxide streaks in this sample were less than 0.1 %. In G1504 and G1806, the laser scanning speed was the main factor in reducing the porosity of the coating. Reducing the scanning speed from 6 mm s^−1^ to 4 mm s^−1^ led to an increase in the interaction of the beam time with the coating, leading to the removal of porosity and oxide streaks in the coating according to the EDS analysis (see Table 9).

**Table 8:** Percentage of oxide and porosity for the NiCrAlY coating before and after laser surface melting.

G(2506)	G(2006)	G(1806)	G(1504)	NiCrAlY-APS	Sample code
> 0.1	1.8 ± 0.4	2.1 ± 0.4	2.3 ± 0.3	8.6 ± 0.8	Porosity

**Table 9:** Analysis of EDS the original coating after 200 h of oxidation from different areas of the oxide layer, as shown in Fig. 18.

Zr	O	Y	Al	Cr	Ni	EDS Spot
2.8 ± 0.4	34.1 ± 0.7	1.4 ± 0.4	6.3 ± 0.4	20.2 ± 0.4	35.2 ± 0.4	Spot 1
7.5 ± 0.6	36.7 ± 0.8	3.8 ± 0.6	3.7 ± 0.4	33.1 ± 0.4	15.2 ± 0.4	Spot 2
2.1 ± 0.4	40.4 ± 0.7	1.6 ± 0.4	45.4 ± 0.6	2.7 ± 0.1	7.8 ± 0.2	Spot 3
6.2 ± 0.6	36.3 ± 0.8	2.3 ± 0.4	1.2 ± 0.4	15.4 ± 0.2	38.6 ± 0.4	Spot 4

**Fig. 17:**
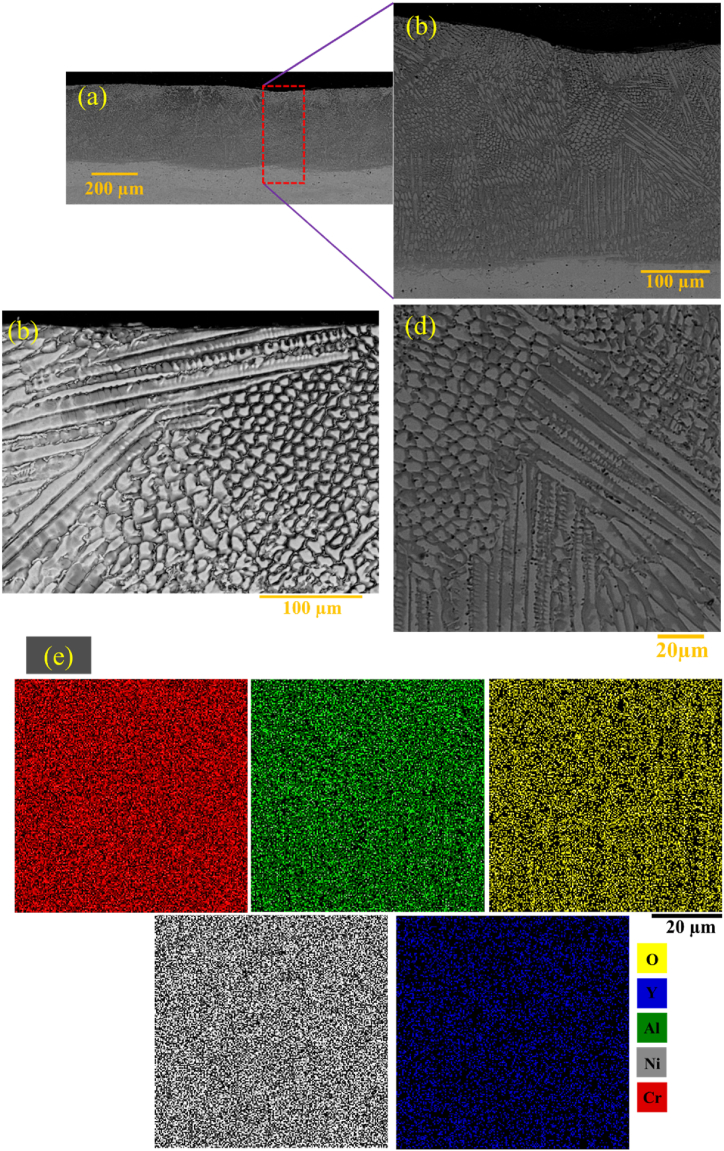
a-d) backscattered electron microscopic image of the microstructure of surface-melted coating related to the sample with the code G2006 in different areas of the coating and e) distribution map of the coating alloy elements of the NiCrAlY coating after laser surface melting.

Fig. 17a–d indicates the backscattered electron microscopy images of the microstructure. Also, Fig. 17e shows the distribution map of the alloy elements of the NiCrAlY coating cross-section after surface melting of G2506 in different areas of the coating. After laser surface melting, the obtained structure consisted of directional grown columnar dendrite (Fig. 17b, d). These results were consistent with those obtained by other researchers [14]. The solidification of the molten coating in contact with the substrate occurs because of heat transfer to the part volume. During laser melting, some positive temperature gradient occurs at the solid-melt interface. For a coating, the solidification microstructure is dependent on some solidification parameters including the solidification rate (R) as well as the temperature gradient of the solid-melt interface (G). As a result, a cellular and dendritic solidification microstructure was formed. These parameters, however, do not measure solidification easily. The G/R value at the bottom of the melt pool is infinite and is decreased throughout the solidification process; while reaching the interface, the cooling speed is increased. In general, throughout laser surface melting, owing to the notable value of the G/R ratio, the solid-melt interface at the bottom of the melt pool is plate-like. Considering the value of G/R, the morphology is changed from a planar one to a cellular one and finally, to a dendritic one. As a result, it leads to the formation of a cellular and dendritic solidification microstructure. The solidification parameters can change quickly at the bottom of the melt pool, although the rate of the change of such parameters declines quickly. Hence, the solidification parameters are almost constant throughout the solidification process. Consequently, it is sometimes difficult to distinguish areas with a plate and cellular microstructure, and the refrozen layer's microstructure may be almost dendritic. The conditions for coaxial growth were provided with the cooling of the melt. Also, due to the presence of porosity and oxide particles in the coating (the presence of solid particles) during melting and solidification, the more feasible conditions are provided for the heterogeneous germination of coaxial crystals. The results, thus, showed that the hardness of the NiCrAlY-APS coating after laser surface melting was influenced by the grain size of the dendrites of the nickel-based solid solution and the decrease of residual stresses, leading to a 9 % drop of microhardness from 305 to 280 Vickers. The results of the adhesion strength of the TBC coating and laser surface fusion coating were obtained to be 41 MPa and 53 MPa, respectively.

Creating a new structure after laser surface melting can be dependent on the speed of cooling and solidification. The presence of porosity in the main coating and the low solidification time caused the formation of a concentric dendritic structure. It, therefore, seemed that laser surface melting led to creating residual stress in the coating.

The high-temperature oxidation test was carried out at a constant temperature of 1100 °C at 50, 100, 150, and 200 h.

**Fig. 18:**
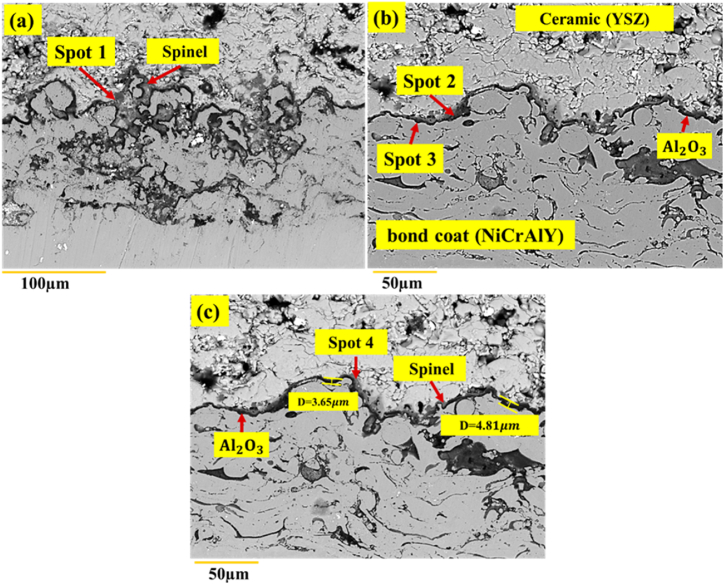
Backscattered electron microscopic images of the TBC-APS sample of the formation of the TGO layer after 200 h a) oxidation test at a temperature of 1100 °C and b, c) changes in the thickness of the TGO layer.

Fig. 18a and b shows the backscattered electron microscopy image of the cross-section of the TBC coating without surface melting after 200 h of the high-temperature oxidation test at 1100 °C. The results, as can be seen in Fig. 18a, showed the roughness of the TGO layer. These surface irregularities are an intrinsic property of APS coatings. As can be seen, the TGO layer was non-uniform and had a different thickness in some areas of Fig. 18c. A dark oxide phase with an average thickness of 3.349 ± 0.23 μm was observed in the interface. Research has shown that in high-temperature coatings, while exposed to high temperatures, a thin oxide layer of Al_2_O_3_ first appears at the interface [30]. After 200 h of oxidation at 1100° Celsius, an Al_2_O_3_ aluminum oxide layer along with harmful NiO, Cr_2_O_3_ and Ni(Cr, Al)_2_O_3_ oxides was formed at the TBC coating's interface without laser surface melting (Table 10). Fig. 18b and c clearly shows this issue. During oxidation, the graft coating roughness had a great effect on the adhesion of the TGO layer. Observations also showed that the thickness difference between the TGO layers was very different for the surface melting coating, as compared to the APS coating. The first factor in the destruction of high-temperature coatings is the growth mechanism. In this respect, the TGO layer's thickness of the TGO layer could considered a measure of the resistance of the coatings against oxidation. On the other hand, the penetration of oxidizing agents in forming the TGO layer could be regarded as a measure of the oxidation rate.

**Table 10:** Changes in the TGO layer thickness for oxidation samples at 1100 °C according to μm.

Sample code				Oxidation time
TBC-APC	G(2006)	G(1806)	G(1504)	
1.9 ± 0.21	1.6 ± 0.18	1.5 ± 0.14	1.3 ± 0.14	50 h
2.3 ± 0.23	1.9 ± 0.2	1.7 ± 0.12	1.4 ± 0.13	100 h
2.5 ± 0.21	2.1 ± 0.22	1.7 ± 0.16	1.5 ± 0.14	150 h
3.3 ± 0.23	2.5 ± 0.2	2.1 ± 0.19	1.7 ± 0.17	200 h

**Fig. 19:**
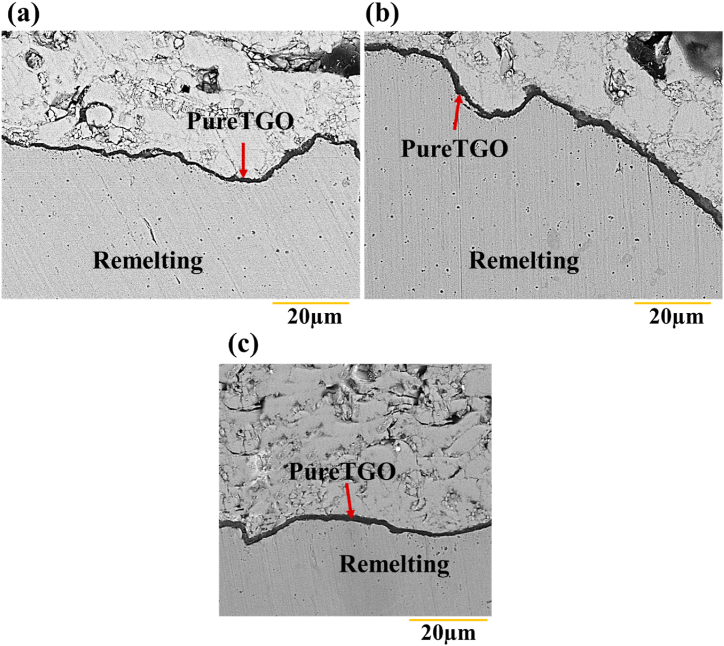
Backscattered electron microscopic images of the thermally grown oxide (TGO) layer in the high temperature oxidation test after 200 h at the temperature of 1100 °C: a) laser surface melting sample with the code G1504, b) laser surface melting sample with the code G1806 and c) Laser surface melting sample with the code G2006.

Fig. 19 a-b and c exhibit the backscattered electron microscopy image of the TGO layer of the laser-melted coating after 200 oxidation of the samples G1504, G1806, and G2004, respectively. At high temperature, preferential oxidation occurred. As oxygen penetrated into the ceramic coating, aluminum moved towards the interface. Fig. 19a shows the backscattered electron microscopy images of the sample G1504. According to the figure, the thickness of the oxide layer in two different areas after 200 h of oxidation was measured to be, on average, 1.686 ± 0.17 μm. The results, thus, showed that because of laser surface melting, the thickness of the TGO layer was decreased, as compared to the sample without surface melting. As heating exceeded 200 h in G1504, a dark-colored layer was formed at the junction of the coating. The oxide layer formed on top of the bonding coating seemed to be the α-Al_2_O rich phase (Fig. 19b and c).

**Fig. 20:**
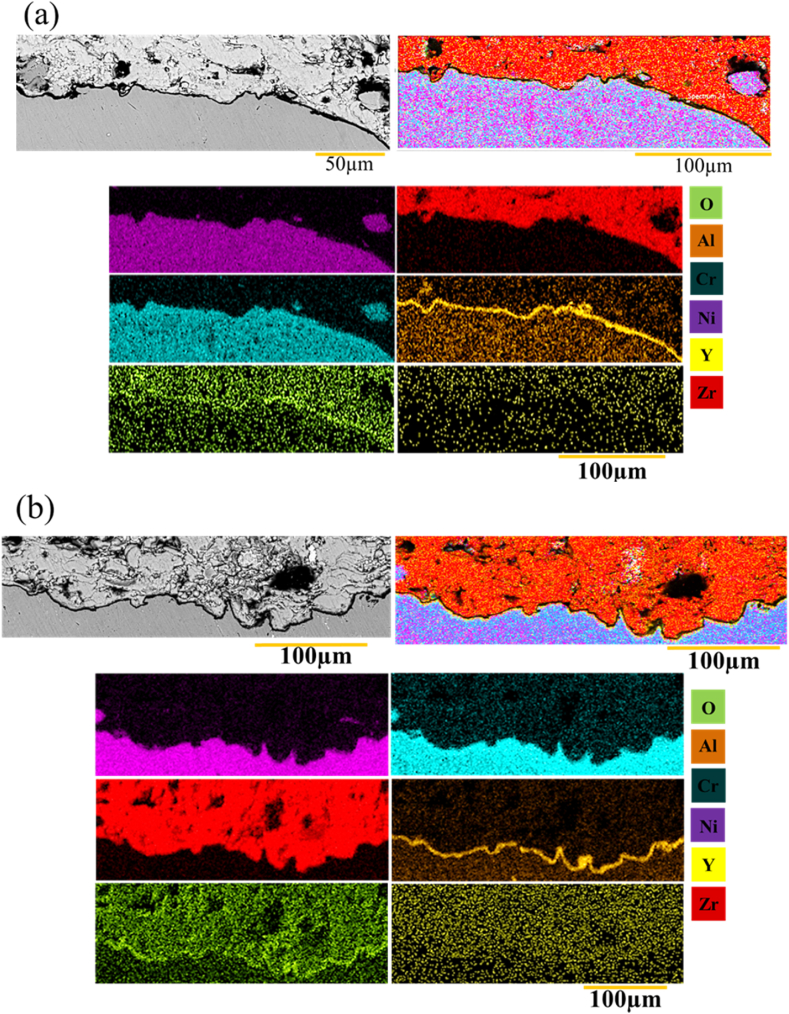

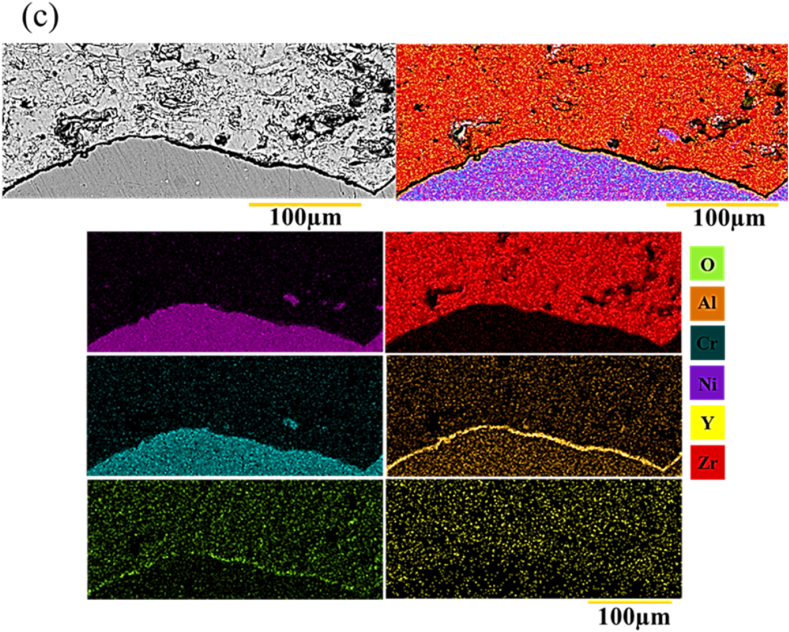
a-c) distribution map of the elements from the interface of the NiCrAlY melting surface coating and ceramic coating, respectively, corresponding to the sample with the codes G1504, G1806 and G2006 after 200 h of oxidation at 1100 °C.

Element distribution map was then used to check the interface in the sample G1504, G1806, G2006. Fig. 20 a-c shows the results of the element distribution map. In this sample, the TGO layer was formed uniformly and continuously, with minimal roughness, thus preventing the growth of harmful oxides on the surface of the interface (Fig. 20 a). Surface melting reduced roughness and prevented excessive aluminum penetration (Fig. 20 b). Therefore, it decreased the thickness of the TGO layer. Aluminum metal is a very reactive material that can quickly react with oxygen because of its high permeability. Roughness was reduced, there was a smaller surface, and thickness of the oxide layer was less. Overall, performing laser surface melting in the bond coating reduced the excessive consumption of aluminum, leading to a TGO layer with lower thickness during oxidation (Fig. 20 c). Moreover, reducing the roughness in the coating prevented the rapid growth of the TGO layer.

**Fig. 21:**
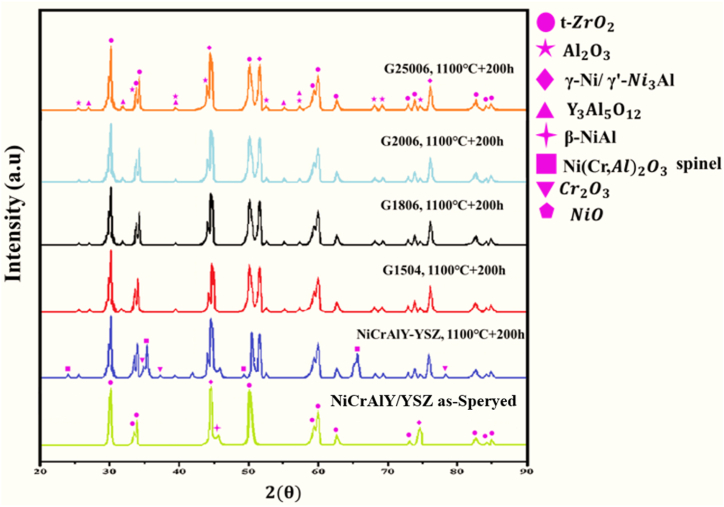
XRD patterns obtained from the oxidation of YSZ/NiCrAlY of the as-sprayed sample and laser melting at 1100 °C for 200 h.

Fig. 21 shows the XRD pattern of the NiCrAlY/YSZ coated sample and laser melting under the high temperature oxidation test for 200 h. The main coating pattern consisted of γꞌ-Ni 3 Al and γ-Ni phase with a small amount of β-NiAl. The lower content of β-NiAl in the bond coating could be due to the oxidation of Al during the sputtering process [31]. Investigations have shown that the displacement of γ/γꞌ and β-NiAl peaks may be due to the stresses caused by the thermal contraction of NiCrAlY spot splats during the spraying processes [32]. ZrO_2_ phase was observed in the ceramic coating. For ceramics, it could be difficult to change its lattice spacing under stress. Peak shift was not observed for ZrO_2_. In the original sample, after 200 h of oxidation, the presence of γNi, NiO, NiCr_2_O_4_ phases, in addition to Al_2_O_3_ and γꞌ-Ni_3_Al phases, was confirmed. The presence of harmful spinel phases in the main coating reduced the oxidation resistance of the coating. β-NiAl phase disappeared in all samples. Also, in the laser melting sample, the decomposition of the β-NiAl phase led to the selective oxidation of Al to Al_2_O_3_ and Y_3_Al_5_O_12_. The presence of Y–Al oxides in the coating acted as a non-penetrating nucleation site enhancing the nucleation of oxides. The grain boundaries of these Y–Al oxides could act as short-circuit diffusion paths for Al ions. The selected parameter was chosen in such a way as to prevent evaporation and the excess consumption of aluminum. The findings obtained from microscopic analysis and X-ray diffraction analysis also showed that laser melting samples reduced the available places for Al2O3 heterogeneous germination by decreasing the height and width of the coating. The laser melting process reduced the heterogeneous storage capacity of the aluminum oxide layer, relative to the original coating. After 200 h of oxidation, a single layer of aluminum oxide was formed.

**Fig. 22:**
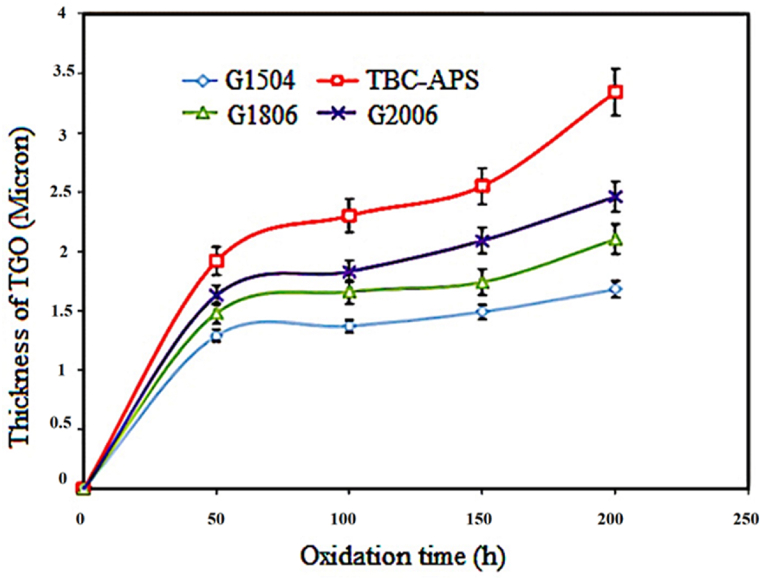
TGO layer thickness change curve in terms of time after the high temperature oxidation test at 1100 °C for TBC coating samples and laser surface melting samples coded G1504, G1806 and G2006.

Fig. 22 depicts the variation curve of the TGO layer thickness according to the oxidation time of 50, 100, 150, and 200 h for the TBC coating and laser surface melting samples with the codes G1504, G1806, G2006, and G2506. Each measured point on the considered curve represents the average of 10 TGO layer thicknesses. The thickness obtained in the G2506 sample was about 20 μm due to the growth of harmful oxides. The kinetics of the formation and growth of the oxide layer in all samples was parabolic. As can be seen, at the beginning of the oxidation test, the TGO layer thickness increased over time. In the following, the growth rate of the TGO layer was decreased in longer oxidation times. The formation of the TGO layer could be attributed to the preferential oxidation during exposure to high temperatures, which depends on the concentration as well as diffusion coefficient. Besides, the diffusion coefficient is dependent on temperature and time. In some laser surface melting samples, the temperature during the process is high. Due to the high speed of the process and the application of Ar shielding gas, it is impossible to form the TGO layer. Given the rotation of the melt, the elements yttrium and aluminum reacted with oxygen. A TGO monolayer with different thicknesses was formed at the TBC coating interface without laser surface melting after 200 h of oxidation. In the sample G1504, a protective oxide monolayer was created at the interface of the coating. In this sample, the TGO layer had a lower and more uniform thickness than the sample without laser surface melting.

In G2506, the TGO layer's growth rate was increased with the prolongation of the oxidation test time. This sample's growth and increase in harmful oxides could be due to a sharp decrease in aluminum activity. In contrast to the samples wherein the bond coating was partially melted on the surface, an oxide layer with minimum thickness was formed. This has also been reported in a similar study [33]. At the time of laser surface melting, the surface-to-volume ratio was decreased in G1504, G1806, and G2006 samples. Accordingly, during oxidation, the rapid consumption of aluminum in these samples could be prevented; as well, the TGO layer's growth was decreased, as compared to the TBC-APS sample. Furthermore, the absence of spinel in the TBC-APS coating, as compared to the G1806, G2006 and G2506 samples, increased the oxidation resistance of this coating, as compared to the lysis of other surface melting coatings. Among the surface-melted samples, the G1504 sample had the minimum thickness and, as a result, displayed higher oxidation resistance than other surface-melted samples. Table 10 quantitatively shows the changes in the TGO layer thickness for the samples G1504, G1806, G2006, G2506, and TBC coating without surface melting. The measured thicknesses were obtained to be from an average of 20 different coating areas. ImageJ 1.52v image analysis software was also applied to measure the TGO layer's thickness. Observations showed that the thickness difference between the TGO layers was very different for the surface melting coating, as compared to the APS coating. The first factor in the destruction of high-temperature coatings is the growth mechanism. Thus, the TGO layer's thickness can be considered a measure of the resistance of the coatings against oxidation. Besides, the penetration of oxidizing agents in creating the TGO layer could be regarded as an oxidation rate measure.

## 4 Conclusion

This research focused on the optimization of the parameters of the laser surface melting process, i.e., the power and speed of laser scanning, to obtain high-quality coatings for specific applications.

• NiCrAlY-APS metal bond coating showed surface roughness and the presence of oxide streaks and porosity in the coating at about 8.6 ± 1.2 %. The surface roughness of the bond coating was 8.13, 13.89, and 22.26 μm for Ra, Rq, and Rz, respectively. Further, the laser surface melting in the NiCrAlY coating reduced the surface's roughness, unevenness, and non-uniformity. It also led to uniformity in the chemical composition of the coating.
• Reducing the thickness and depth of a single pass depends on the scanning speed as well as laser power. At constant power, the depth of surface melting coating was increased with raising laser interaction duration. As a result, using a laser power 250 W with a scanning speed of 4 mm s^−1^ in a multi-pass manner caused a significant decrease of porosity and oxide in the NiCrAlY-APS coating from 8.6 ± 1.2 % to less than 0.1 %.
• Laser surface melting of NiCrAlY coating in a multi-pass manner caused the coating elements to rotate during melting, and light aluminum and yttrium elements were scattered on the top surface of the coating. After melting the laser surface, the structure consisted of oriented columnar dendrites.
• In the interface of the original coating after 200 h of oxidation, an Al_2_O_3_ aluminum oxide layer was formed along with harmful NiO, Cr_2_O_3_ and Ni(Cr, Al)_2_O_3_ with the thickness of 3.349 ± 0.23 μm at the coating interface.
• The surface melting of NiCrAlY metallic bonding coating in TBC coatings led to a decrease in the coating's surface area. Thus, the thickness of the TGO layer declined during high-temperature oxidation. The surface melting sample G1504 developed a higher oxidation resistance than other samples due to the creation of a dense and protective TGO single layer between the junction of the bonding coating and the ceramic coating with the thickness of 1.686 ± 0.17 μm.
• The surface-melted sample G1504, as compared to other surface-melted samples, created a good and higher oxidation resistance owing to forming a single protective oxide layer of TGO.
• Laser melting of the NiCrAlY-APS interlayer led to the reduction of surface roughness and the available places for Al_2_O_3_ heterogeneous nucleation. As a result, by reducing the storage capacity of the aluminum oxide layer, as compared to the main coating, a higher resistance to oxidation could be seen.

## Data availability statement

The authors do not have permission to share data.

## CRediT authorship contribution statement

**Mohammad Gavahian Jahromi:** Writing – review & editing, Writing – original draft, Software, Resources, Project administration, Methodology, Investigation, Formal analysis, Data curation, Conceptualization. **Reza Shoja Razavi:** Methodology. **Zia Valefi:** Software. **hamed Naderi:** Data curation. **Saeid Taghi-Ramezani:** Validation, Software.

## Declaration of competing interest

The authors declare that they have no known competing financial interests or personal relationships that could have appeared to influence the work reported in this paper.
